# Effect of Deep Cryogenic Activated Treatment on Hemp Stem-Derived Carbon Used as Anode for Lithium-Ion Batteries

**DOI:** 10.1186/s11671-020-03422-w

**Published:** 2020-10-01

**Authors:** Zhigang Li, Zhongxiang Guan, Zhiping Guan, Ce Liang, Kaifeng Yu

**Affiliations:** 1 Key Laboratory of Automobile Materials, Ministry of Education, College of Materials Science and Engineering, Jilin University, Changchun, 130025 China; 2 Institute of Superplastic and Plastic of Jilin University, Changchun, 130025 China

**Keywords:** Cryogenic process, Hemp stems, Pore structure, Lithium-ion batteries, High specific capacity

## Abstract

The cryogenic process has been widely applied in various fields, but it has rarely been reported in the preparation of anode materials for lithium-ion battery. In this paper, activated carbon derived from hemp stems was prepared by carbonization and activation; then, it was subjected to cryogenic treatment to obtain cryogenic activated carbon. The characterization results show that the cryogenic activated carbon (CAC) has a richer pore structure than the activated carbon (AC) without cryogenic treatment, and its specific surface area is 1727.96 m^2^/g. The porous carbon had an excellent reversible capacity of 756.8 mAh/g after 100 cycles at 0.2 C as anode of lithium-ion battery, in which the electrochemical performance of CAC was remarkably improved due to its good pore structure. This provides a new idea for the preparation of anode materials for high-capacity lithium-ion batteries.

## Introduction

Due to agricultural waste, such as rice husks, stems, and fibers, having the advantages of rich resources and reproducibility, researchers have paid great attention to the development and application of these agricultural wastes, which are not usually noticeable. Nowadays, there have been many innovative advances in the research and application of biomass carbon materials, providing good theoretical support for preparation of the high-quality anode materials for lithium-ion batteries. Many researchers are constantly trying new biomass carbon sources and treatment processes to improve the quality of biomass carbon and apply it to different fields. As a traditional treatment method, activation treatment can effectively improve the porosity of the material and increase the active site [1–5]. Pan et al. used K_2_FeO_4_ to complete the simultaneous carbonization and graphitization of bamboo charcoal, which takes less time and has high efficiency [1]. In the treatment of biomass carbon, hydrothermal methods are more and more widely used in current production and scientific research [6–11]. Yang et al. extracted hemicellulose from hemp stem and prepared it into a well-shaped carbon sphere by low-temperature hydrothermal and KOH activation, which is a potential sustainable material for energy and environmental applications [6]. The structural size of biomass carbon can be more accurately and effectively controlled by the template method. In addition, the template method has huge advantages in controlling the size of the material and has great application prospects [12–15]. Lin et al. prepared a hierarchical porous hard carbon from rubber wood sawdust via a ZnO-based hard template method and applied it to sodium ion batteries [12]. Although the current research methods of biomass carbon materials are approaching maturity, the development of new process methods and new materials is still the development direction of electrode materials [16–20].

Cryogenic process is a new type of material processing technology, and more widely used in the metal field currently. Cryogenic treatment can refine metal crystal size to achieve excellent mechanical properties [21–24]. Abrosimova et al. investigated the effect of cryogenic treatment on the rejuvenation of the amorphous phase of Al-based alloys [21]. Li et al. explored the effect of cryogenic treatment (CT) on the mechanical properties and microstructure of IN718 superalloy [22]. Cryogenic treatment also has excellent applications in the fields of composite materials and fibers [25–32]. Shao et al. explored the effects of low-temperature treatment on the interfacial characteristics and electrical resistance of carbon nanotube (CNT) fiber/epoxy composites [25]. In addition, cryogenic treatment has also made achievements in other fields [33–35]. Song et al. summarized the characteristics of cryogenic technologies for CO_2_ capture [33]. Guo et al. evaluated the effects of various experimental conditions on the regeneration behavior of Zr-based metallic glass during deep cryogenic cycling treatment [35]. Cryogenic treatment has an extraordinary role in many fields and is reasonably applied, but there are few reports on the treatment of biomass carbon materials and applied it into lithium-ion battery.

In this paper, cryogenic treatment process, a purely physical treatment method, is applied to improve the quality of activated carbon, so that it can form more pores that are widened and make the overall structure relatively stable, which is beneficial to improve subsequent electrochemical performance. Activated carbon material is obtained by activating hemp stems, and then cryogenic treatment to further widen the pore size, stabilize the carbon structure, and change the physical and chemical properties of the material. The obtained cryogenic activated carbon was named CAC and applied into anode for lithium-ion battery, which has a high specific capacity. The method is an ideal preparation way for realizing a low-cost, high-efficiency, high specific capacity anode for lithium-ion battery.

## Materials and Methods

### Preparation of Cryogenic Activated Carbon Derived from Hemp Stems

**Fig. 1:**
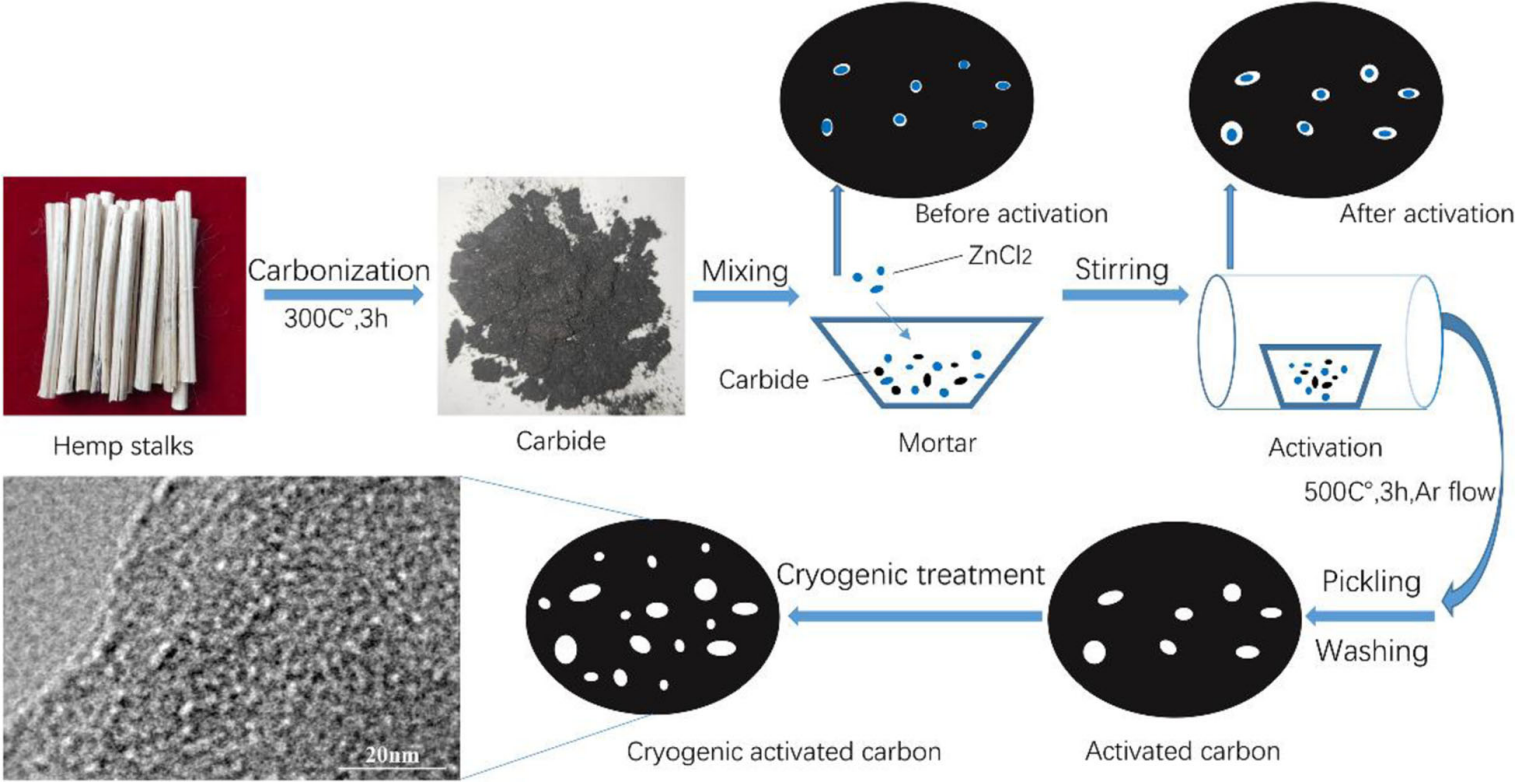
Schematic illustrations for preparing cryogenic activated carbon with porous structure

**Fig. 2:**
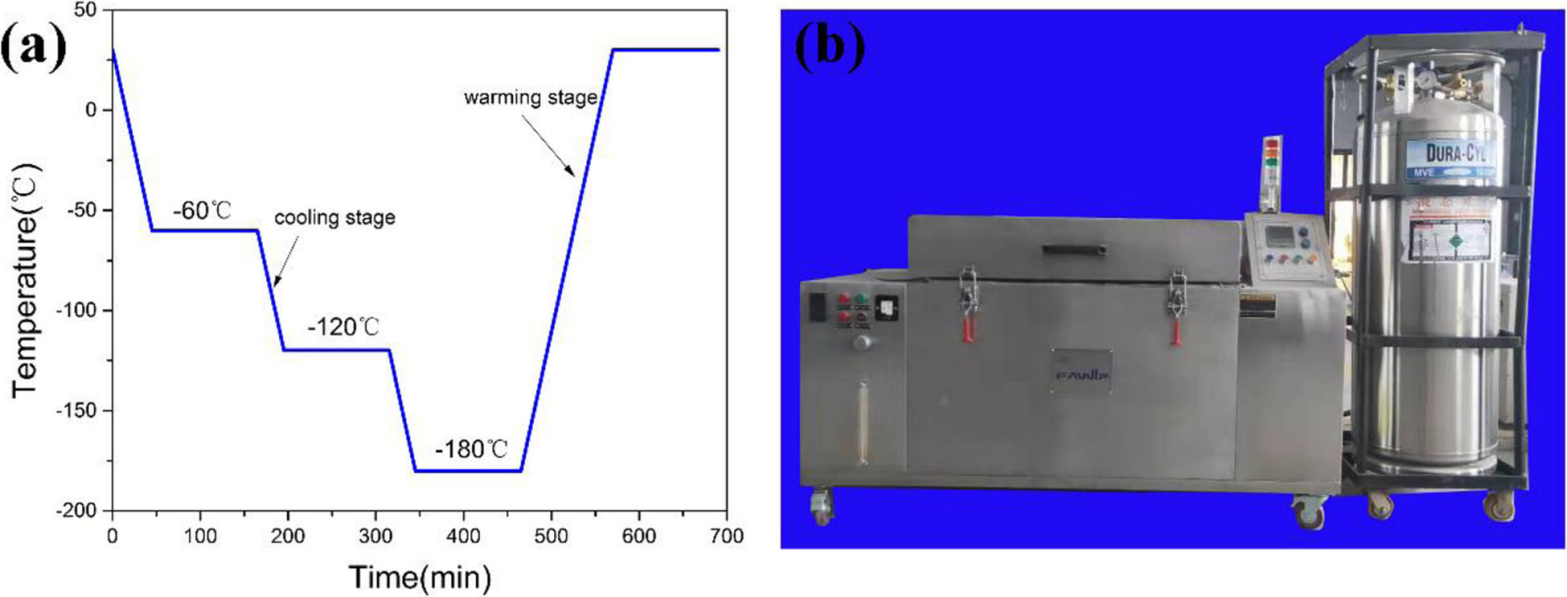
**a** Process curve of the cryogenic treatment. **b** Program-controlled cryogenic chamber

Hemp stems were gained from the field of Heilongjiang Province, China. As shown in the schematic diagram of Fig. 1, the activated carbon were prepared using the method [36] which was mass ratio of 1:5 and the mixture temperature of 500 °C. The dried activated carbon was placed in a cryostat and cooled gradually to − 185 °C for 2 h, as shown in Fig. 2. Then, it is returned to room temperature to obtain cryogenic activated carbon material. Cryogenic activated carbon samples were denoted as CAC-*β*, where *β* is the activation temperature. The sample that was also activated at 500 °C without undergoing cryogenic treatment was denoted as AC-500.

### Materials Characterization

The microstructure of the activated carbon was observed by field emission scanning electron microscope (JEOL JSM-6700F) and transmission electron microscope (JEM-2100F). The X-ray diffraction (XRD) pattern of the hemp stems powder was observed by Siemens D5000 X-ray Diffractometer. The specific surface area and pore size distribution of carbon material were measured by nitrogen adsorption-desorption measurement (Micromeritics, ASAP2420). The Raman spectra were observed with Renishaw inVia instrument.

### Electrochemical Measurements

Using the cryogenic activated carbon, the button battery was prepared using the methods [36]. After assembly, the cycle performance test of the button battery was performed by LAND battery test system at the voltage range of 0.02~3 V. The cyclic voltammetry (CV) curve and impedance test was performed on the electrochemical workstation.

## Results and Discussion

### Structural and Morphological Characterization

**Fig. 3:**
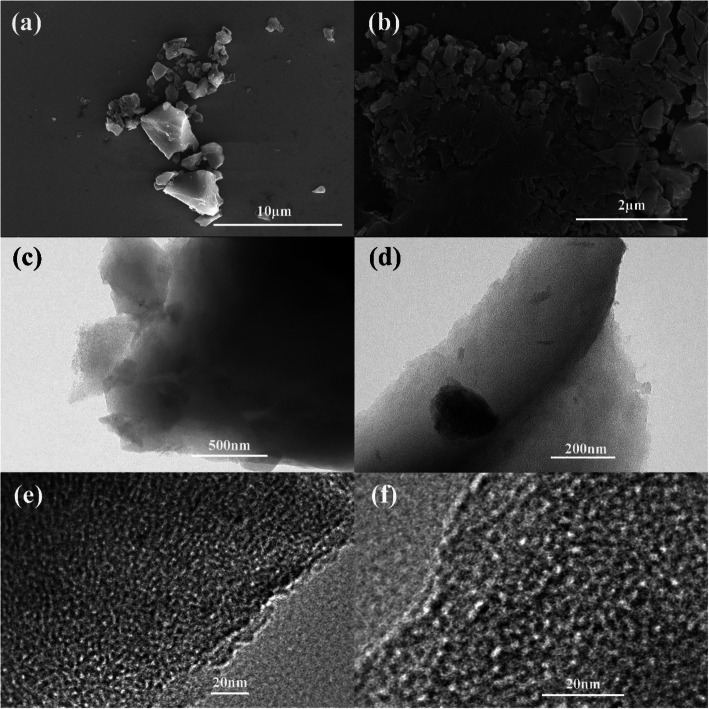
**a** SEM image of AC-500. **b** SEM image of CAC-500. **c** TEM pattern of AC-500. **d** TEM pattern of CAC-500. **e** HRTEM pattern of AC-500. **f** HRTEM pattern of CAC-500

The activated carbon derived from hemp stems is obtained through carbonization and activation pretreatment as shown in Fig. 3a. After the cryogenic treatment, the morphology of CAC-500 did not undergo other changes overall, except that it was more fragmented, as shown in Fig. 3b, which is due to the brittleness of AC-500 increased and cracking occurred by cryogenic treatment. The fragmented material can be provided more active sites because of a large number of sheet-like structures and slit-like interspace. Both are amorphous carbon overall, and no obvious macropores are observed. At large magnifications, AC-500 and CAC-500 have rich pore structures, and most of them are microporous or mesoporous, which will facilitate the storage and transmission of lithium ions as shown in Fig. 3e and f.

**Fig. 4:**
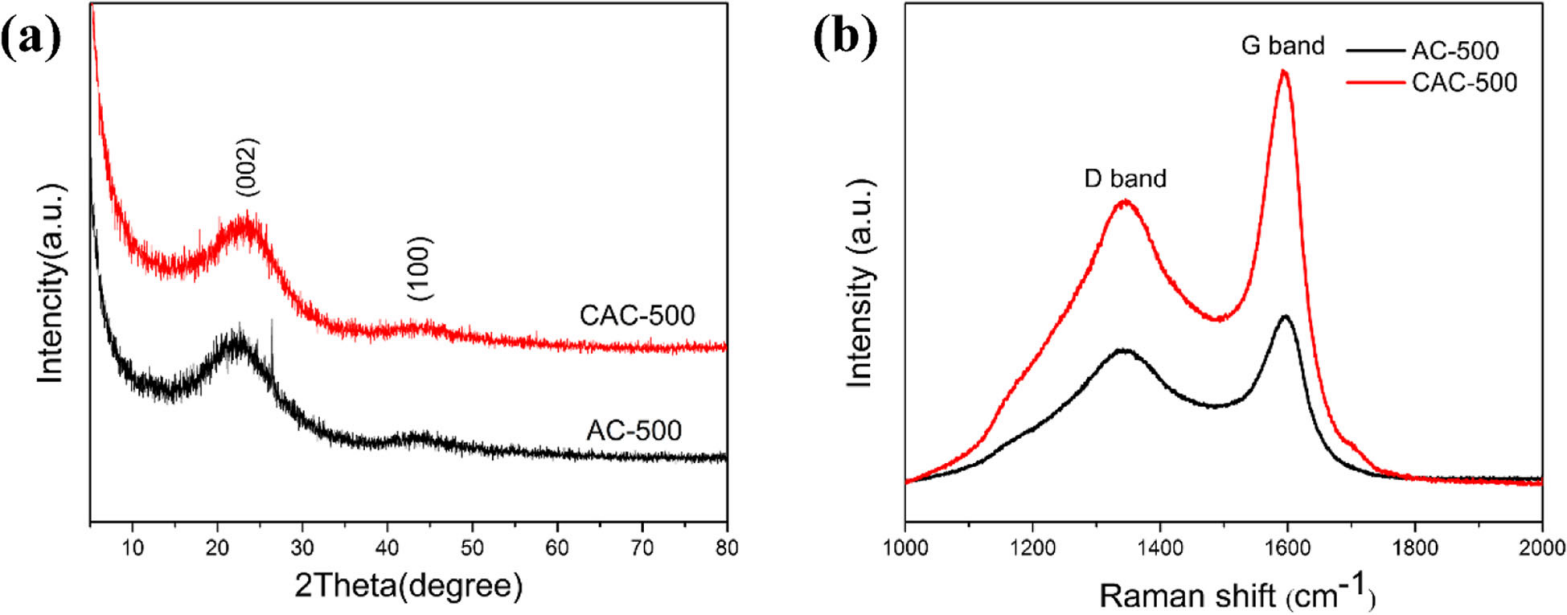
**a** X-ray diffraction patterns. **b** Raman spectrums of AC-500 and CAC-500

The X-ray diffraction patterns of the two samples obtained before and after cryogenic are shown in Fig. 4a. It is obvious that there are two distinct diffraction peaks at 22° and 44°, corresponding to the (002) and (100) crystal planes of the graphite structure, respectively. The 22° diffraction peak is due to the presence of continuous parallel graphite flakes, while the 44° diffraction peak is caused by the honeycomb structure formed by sp2 hybridization. In addition, both samples exhibit the characteristics of traditional amorphous carbon materials due to the absence of sharp diffraction peaks.

The Raman spectra of AC-500 and CAC-500 are shown in Fig. 4b. The tow materials have evident D peak and G peak. The D peak is induced by the defects of the material, while the G peak is generated by the vibration of the sp^2^ hybrid carbon atoms of the graphite sheet. The intensity ratio of D peak to G peak is usually used to characterize the degree of material defects. Accordingly, the calculated ratios of AC-500 and CAC-500 are 0.7937 and 0.6899. It indicates that the two materials have high amorphousness and more edges and defects, which can provide more active sites for the insertion of lithium ions, thereby exhibiting preeminent electrochemical performance.

**Fig. 5:**
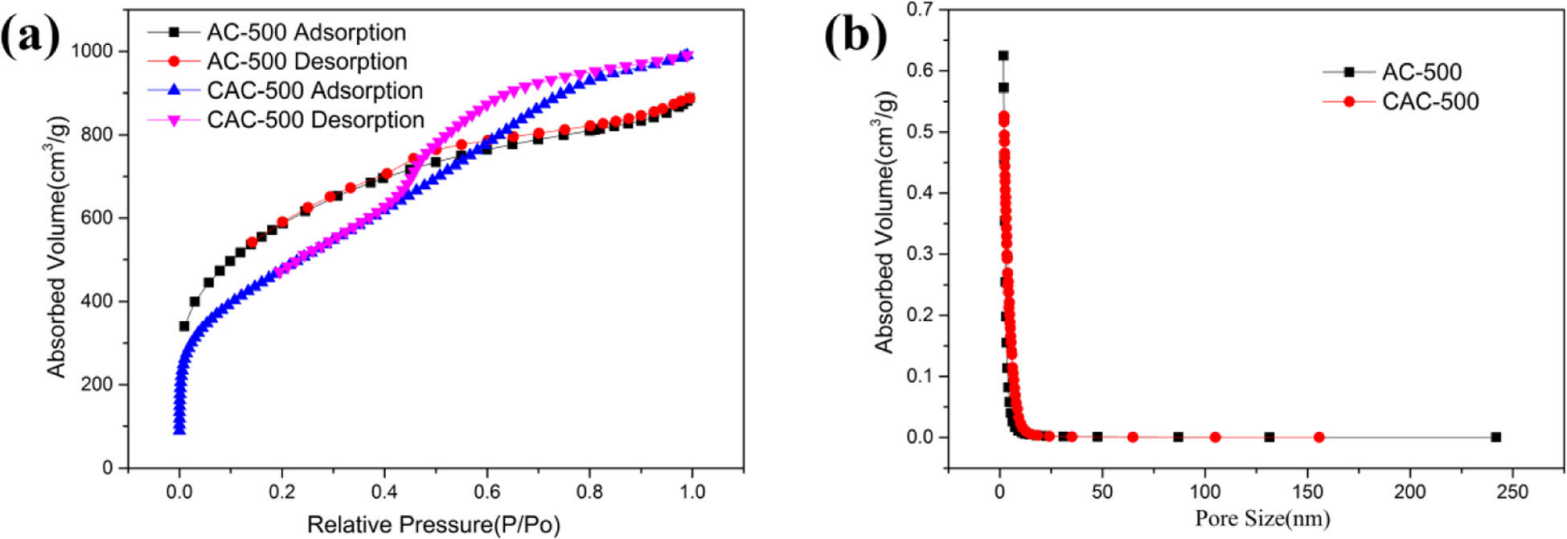
**a** Isothermal adsorption-desorption curve of AC-500 and CAC-500. **b** Pore size distribution of AC-500 and CAC-500

Figure 5 displays the specific surface area and pore size distribution of the two materials. The specific surface area of AC-500 and CAC-500 are 2024 m^2^/g and 1728 m^2^/g, respectively. The lower specific surface area indicates that the CAC-500 material has more macropores and mesopores, which will improve the efficiency of cycling insertion and extraction of lithium ion [37]. At the same time, the corresponding average adsorption pore size of AC-500 and CAC-500 are 2.651 nm and 3.547 nm. The isotherm adsorption and desorption curve in Figure 5a shows that the types of AC-500 and CAC-500 are type I and type IV, and the types of closed hysteresis loop are H4 and H1, respectively. Obviously, it is confirmed that AC-500 has more microporous structures, while CAC-500 has a large number of mesoporous structures. In addition, the CAC-500 sample reflects the cylindrical hole with a uniform diameter at both ends, which can be realized with mesoporous materials with a relatively narrow pore size distribution.

### Electrochemical Characterization

**Fig. 6:**
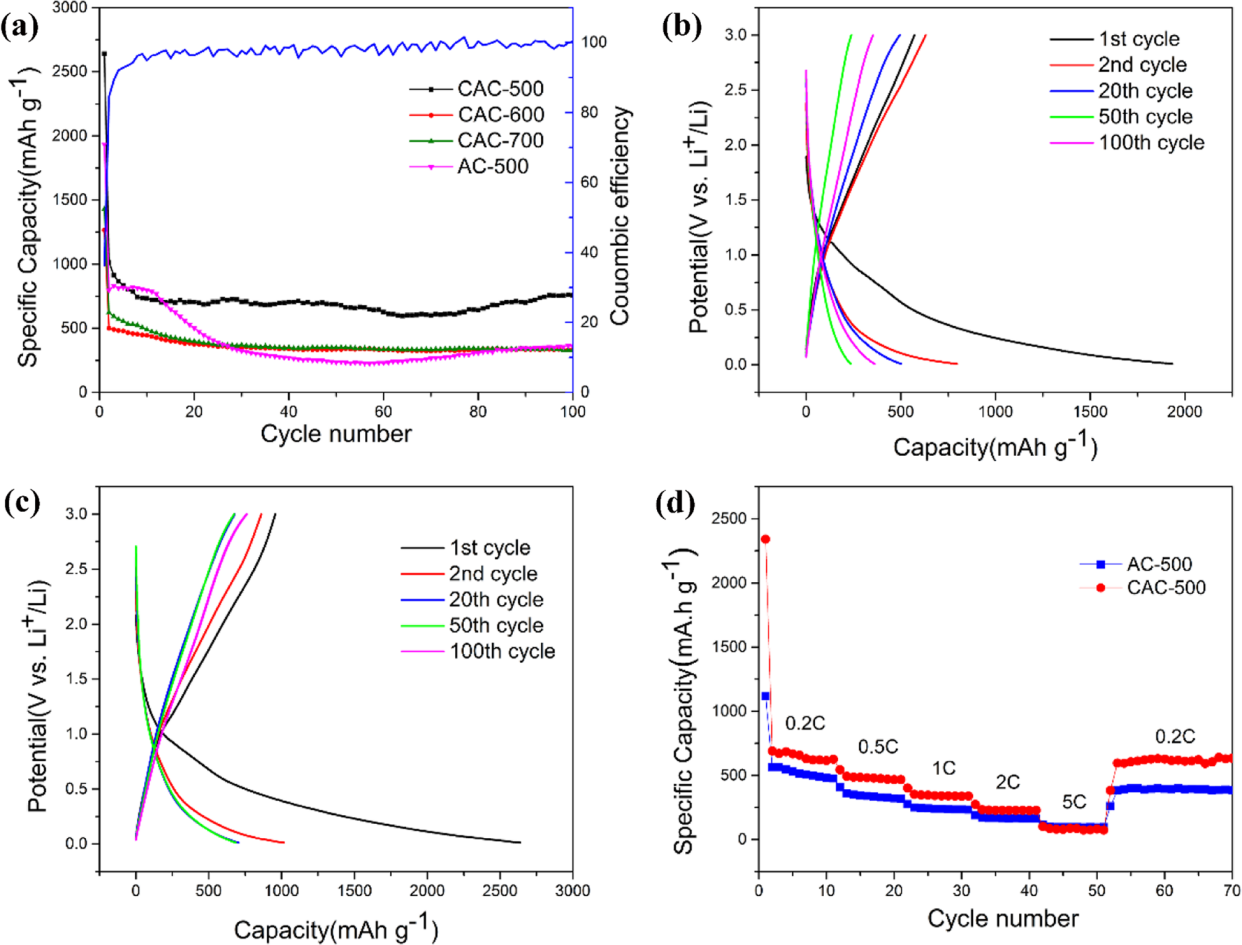
**a** Cycle performance curves. **b** Charge-discharge voltage curves of AC-500. **c** Charge-discharge voltage curves of CAC-500. **d** Rate performance of AC-500 and CAC-500

Figure 6a shows that the charge-discharge cycle performance of cryogenic activated carbon by different activation temperatures at a rate of 0.2 C, in which the current corresponding to 1 C is 372 mA. Clearly, CAC-500 exhibits an excellent cycling performance of 740 mAh/g. Compared with CAC-600 and CAC-700, CAC-500 performs a better cycle performance that are stems from the abundant mesoporous and microporous structures inside the material. The first discharge specific capacity and charge specific capacity of CAC-500 are 2469.7 mAh/g and 1168.1 mAh/g, respectively. The relatively poor coulomb efficiency of first cycle (only about 36%) is in a good agreement with the common characteristics of lithium-ion batteries cycle performance [38, 39]. It is the large amount of lithium ions consumed by the solid electrolyte interface (SEI) film formed in the first cycle due to the large specific surface area that leads to the huge capacitance loss of the first cycle. In addition, its other coulomb efficiency is around 100%, indicating that AC-600 has a small capacity loss rate. Figure 6b and c show the charge and discharge curves from the first cycle to the 100th cycle of AC-500 and CAC-500, where the charge and discharge curves gradually became consistent as the increasing number of cycles. The discharge curves of CAC-500 at the 20th, 50th, and 100th cycles almost completely coincide, while the AC-500 possesses a lower degree of coincidence and exhibits the unstable electrochemical performance, implying the better stability of CAC-50 in electrochemical performance.

Figure 6d plots the rate discharge performance of the as-prepared materials at current densities of 0.2–5 C. The good rate ability can be observed for CAC-500 with average discharge capacities of 615.7 mAh/g, 467.1 mAh/g, 336.9 mAh/g, 225.4 mAh/g, and 80.6 mAh/g at current densities of 0.2 C, 0.5 C, 1 C, 2 C, and 5 C, separately. It is noteworthy that the initial performance of the AC-600 is high although the capacity drops significantly at large magnifications. However, the performance of CAC-500 can still be restored to a higher reversible capacity of 627 mAh/g when the discharge rate is restored to 0.2 C, indicating the better capacity retention of CAC-500. Conversely, the lower rate performance capacity of AC-500 is exhibited with the average discharge capacities of 480.7 mAh/g, 320.8 mAh/g, 233.8 mAh/g, 162.4 mAh/g, 95 mAh/g, and 394.1 mAh/g at same current densities as CAC-500, which is due to the increase of active sites and the expansion of pore structure caused by cryogenic treatment.

**Fig. 7:**
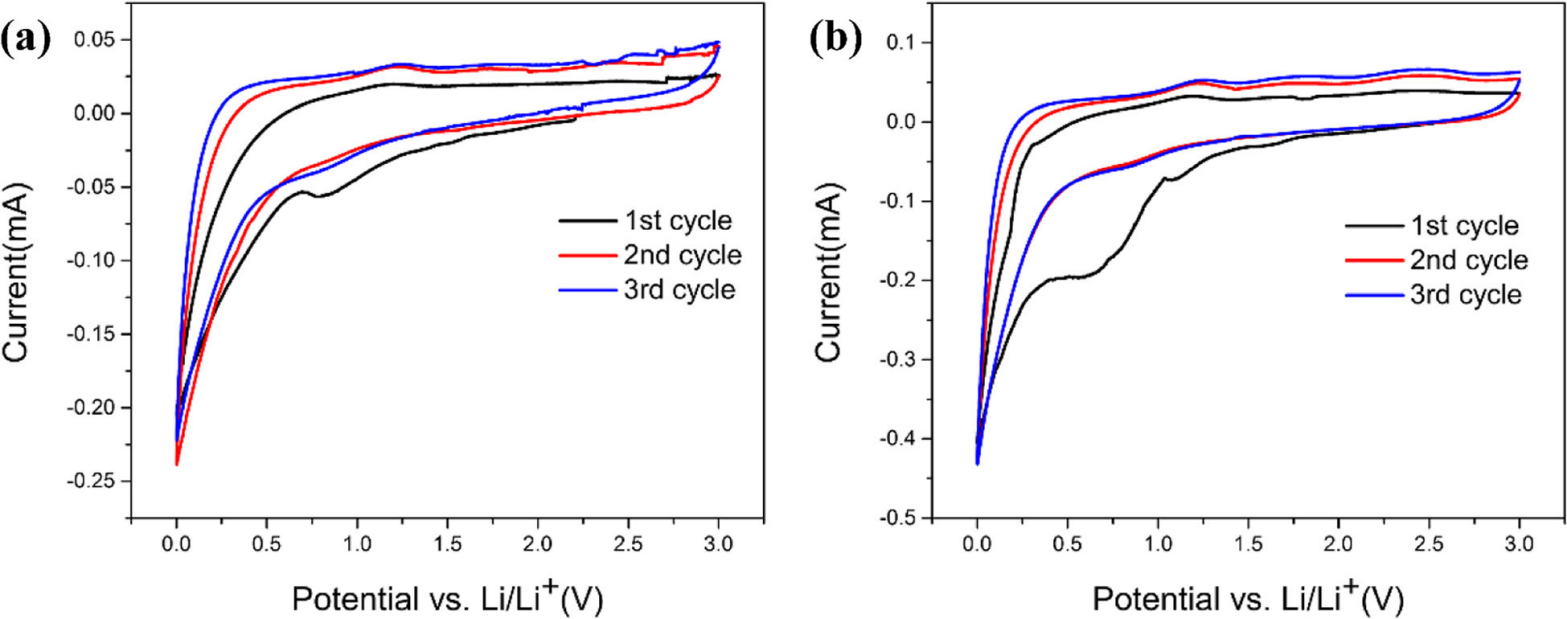
**a** Cyclic voltammogram profiles of AC-500. **b** Cyclic voltammogram profiles of CAC-500

Figure 7a and b exhibit the initial three cycles of cyclic volt-ampere (CV) curves at a scan rate of 0.1 mV/s between 0.01 and 3.0 V. Clearly, there exist a sharp peak around 0.7 V and a weak peak around 1.35 V in the reduction process of the first circle, indicating that an irreversible reaction has begun between electrode and electrolyte [40]. Note that it is the decomposition of the electrolyte on the electrode surface and the formation of the SEI film that lead to the forming of the peak around 0.7 V. The vanishment of these peaks in the subsequent second and third cycle is due to the irreversible reactions in the first cycle. In the first cycle, lithium deintercalation process occurs at anodic peak around 0.25 V, which is consistent with the reported carbon substance [1, 40]. Both AC-500 and CAC-500 have a tendency to gradually coincide with the subsequent second and third cycles, and the second and third circles are completely coincident in Fig. 7, indicating the good stability of electrode material.

**Fig. 8:**
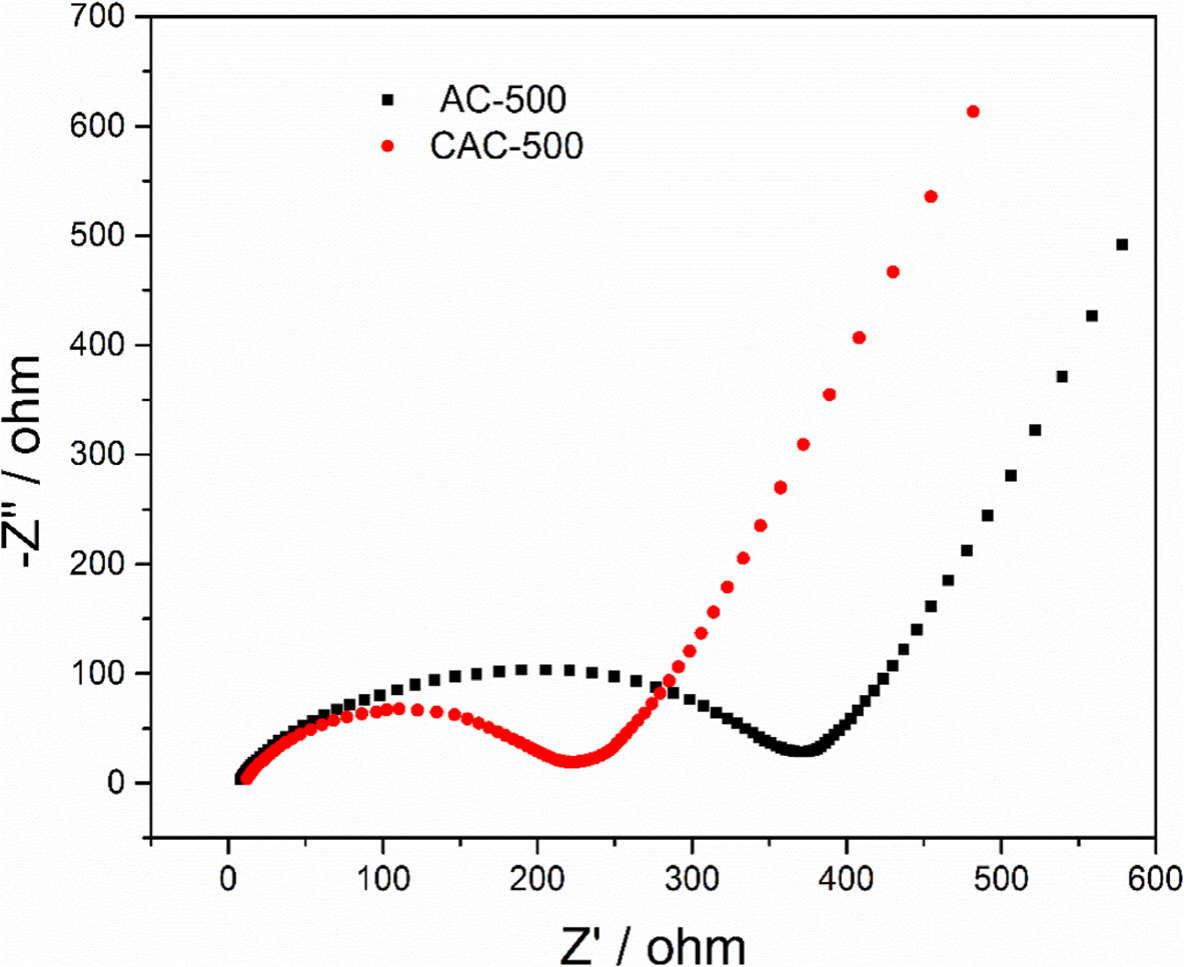
Impedance spectrums of AC-500 and CAC-500

We also tested the impedance spectrum of AC-500 and CAC-500 to further demonstrate the kinetics of the electrodes during lithium ion transport, as shown in Fig. 8. The larger contact resistance of AC-500 than that of CAC-500 can be explained by the difference of high frequency region. Although there is no remarkable difference in charge transfer impedance corresponding to the IF region, the diffusion impedance corresponding to high frequency region of CAC-500 is significantly smaller than that of AC-500. These results demonstrate that the AC-500 after cryogenic treatment has a small impedance, which is due to more mesopores produced by the activated carbon after cryogenic production, thereby reducing the diffusion resistance of lithium ions.

## Conclusions

The activated carbon derived from hemp stems has a rich pore structure, and the vast majority of the pores is microporous. Further, the cryogenic treatment of the activated carbon not only widens the pore diameter of the material, but also produces more mesopores, which reduces the impedance and improves the electrochemical performance. Cryogenic activated carbon has a high surface area of 1728 m^2^/g and an excellent specific capacity of 756.8 mAh/g, making it an ideal material for anode material of lithium-ion battery. The preparation of cryogenic activated carbon derived from hemp stems for lithium-ion battery is not only the successful application of hemp stems, but also provides a new idea for the development of anode materials for lithium-ion batteries.

## Data Availability

The conclusions made in this manuscript are based on the data which are all presented and shown in this paper.

## Abbreviations

CAC: Cryogenic activated carbon
AC: Activated carbon
CV: Cyclic voltammetry
SEI: Solid electrolyte interface
DMC: Dimethyl carbonate
EC: Ethylene carbonate
EMC: Ethyl methyl carbonate

## References

[1] Gong Y, Li D, Luo C, Fu Q, Pan C (2017). Highly porous graphitic biomass carbon as advanced electrode materials for supercapacitors. Green Chem.

[2] Yang R, Liu G, Li M, Zhang J, Hao X (2012). Preparation and N_2_, CO_2_ and H_2_ adsorption of super activated carbon derived from biomass source hemp (*Cannabis sativa* L.) stem. Microporous Mesoporous Mater.

[3] Wang L, Sun F, Hao F, Qu Z, Gao J, Liu M, Wang K, Zhao G, Qin Y (2020). **A** green trace K_2_CO_3_ induced catalytic activation strategy for developing coal-converted activated carbon as advanced candidate for CO_2_ adsorption and supercapacitors. Chem End J.

[4] Lin H, Liu Y, Chang Z, Yan S, Liu S, Han S (2020). A new method of synthesizing hemicellulose-derived porous activated carbon for high-performance supercapacitors. Microporous Mesoporous Mater.

[5] Pena J, Villot A, Gerente C (2020). Pyrolysis chars and physically activated carbons prepared from buckwheat husks for catalytic purification of syngas. Biomass Bioenergy.

[6] Wang Y, Yang R, Li M, Zhao Z (2015). Hydrothermal preparation of highly porous carbon spheres from hemp (*Cannabis sativa* L.) stem hemicellulose for use in energy-related applications. Ind Crop Prod.

[7] Xu Y, Zhang C, Kuang S, Zhao K, Chen M, Xu D, Chen W, Yu X (2019). Facile synthesis and electrochemical performances of activated bamboo charcoal supported MoS_2_ nanoflakes as anodes materials for lithium-ion batteries. J Electroanal Chem.

[8] Mbarki F, Selmi T, Kesraoui A, Seffen M, Gadonneix P, Celzard A, Fierro V (2019). Hydrothermal pre-treatment, an efficient tool to improve activated carbon performances. Ind Crop Prod.

[9] Liu B, Liu S, Meng L, Li Y, Wang B, Ma M (2018). Microwave-hydrothermal synthesis and photocatalytic properties of biomass charcoal/TiO2 nanocomposites. J Saudi Chem Soc.

[10] Yadav MS (2020). Synthesis and characterization of Mn_2_O_3_−Mn_3_O_4_ nanoparticles and activated charcoal based nanocomposite for supercapacitor electrode application. J Energy Storage.

[11] Wei H, Wang X, Zhang D, Du W, Sun X, Jiang F, Shi T (2019). Facile synthesis of lotus seedpod-based 3D hollow porous activated carbon/manganese dioxide composite for supercapacitor electrode. J Electroanal Chem.

[12] Muruganantham R, Hsieh T, Lin C, Liu W (2019). Bio-oil derived hierarchical porous hard carbon from rubber wood sawdust via a template fabrication process as highly stable anode for sodium-ion batteries. Mater Today Energy.

[13] Xiao P, Zhao L, Sui Z, Xu M, Han B (2017). Direct synthesis of ordered mesoporous hydrothermal carbon materials via a modified soft-templating method. Microporous Mesoporous Mater.

[14] Luo J, Zhang H, Zhang Z, Yu J, Yang Z (2019). In-built template synthesis of hierarchical porous carbon microcubes from biomass toward electrochemical energy storage. Carbon..

[15] Yuan H, Chen J, Li D, Chen H, Chen Y (2019). 5 Ultramicropore-rich renewable porous carbon from biomass tar with excellent adsorption capacity and selectivity for CO_2_ capture. Chem Eng J.

[16] Sheng S (2020). Zhang, Identifying rate limitation and a guide to design of fast-charging Li-ion battery. InfoMat..

[17] Liang Y, Zhao C-Z, Yuan H, Chen Y, Zhang W, Huang J-Q, Yu D, Liu Y, Titirici M-M, Chueh Y-L, Yu H, Zhang Q (2019). A review of rechargeable batteries for portable electronic devices. InfoMat..

[18] Zhao Y, Guo J (2020). Development of flexible Li-ion batteries for flexible electronics. InfoMat..

[19] Dong C, Tan H, Rui X, Zhang Q, Feng Y, Geng H, Li C, Huang S, Yu Y (2019). Oxyvanite V3O5: A new intercalation-type anode for lithium-ion battery. InfoMat.

[20] Zeng Z, Liu X, Jiang X, Liu Z, Peng Z, Feng X, Chen W, Xia D, Ai X, Yang H, Cao Y (2020). Enabling an intrinsically safe and high-energy-density 4.5 V-class Li-ion battery with nonflammable electrolyte. InfoMat.

[21] Abrosimova G, Volkov N, Pershina E, Tuan TV, Aronin A (2020). Amorphous structure rejuvenation under cryogenic treatment of Al-based amorphous-nanocrystalline alloys. J Non-Cryst Solids.

[22] Li J, Zhou J, Xu S, Sheng J, Huang S, Sun Y, Sun Q, Boateng EA (2017). Effects of cryogenic treatment on mechanical properties and micro- structures of IN718 super-alloy. Mat Sci Eng A.

[23] Aghamiri SMS, Zhang SH, Ukai S, Oono N, Kasada R, Noto H, Hishinuma Y, Muroga T (2020). Microstructure development in cryogenically rolled oxide dispersion strengthened copper. Acta Mater.

[24] Li S, Xiao M, Ye G, Zhao K, Yang M (2018). Effects of deep cryogenic treatment on microstructural evolution and alloy phases precipitation of a new low carbon martensitic stainless bearing steel during aging. Mat Sci Eng A.

[25] Shao Y, Xu F, Liu W, Zhou M, Li W, Hui D, Qiu Y (2017). Influence of cryogenic treatment on mechanical and interfacial properties of carbon nanotube fiber/bisphenol-F epoxy composite. Compos Part B-Eng.

[26] Xu F, Fan W, Zhang Y, Gao Y, Jia Z, Qiu Y, Hui D (2017). Modification of tensile, wear and interfacial properties of Kevlar fibers under cryogenic treatment. Compos Part B-Eng.

[27] Zhang Y, Xu F, Zhang C, Wang J, Jia Z, Hui D, Qiu Y (2016). Tensile and interfacial properties of polyacrylonitrile-based carbon fiber after different cryogenic treated condition. Compos Part B-Eng.

[28] Zhang M, Li K, Shi X, Guo L, Feng L, Duan T (2018). Influence of cryogenic thermal cycling treatment on the thermophysical properties of carbon/carbon composites between room temperature and 1900 °C. J Mater Sci Technol.

[29] Li GR, Cheng JF, Wang HM, Li CQ (2017). The influence of cryogenic-aging circular treatment on the microstructure and properties of aluminum matrix composites. J Alloys Compd.

[30] He Y, Chen Q, Yang S, Lu C, Feng M, Jiang Y, Cao G, Zhang J, Liu C (2018). Micro-crack behavior of carbon fiber reinforced Fe_3_O_4_/graphene oxide modified epoxy composites for cryogenic application. Compos Part A-Appl S.

[31] He Y, Li Q, Kuila T, Kim NH, Jiang T, Lau K, Lee JH (2013). Micro-crack behavior of carbon fiber reinforced thermoplastic modified epoxy composites for cryogenic applications. Compos Part B-Eng.

[32] Morkavuk S, Köklü U, Bağcı M, Gemi L (2018). Cryogenic machining of carbon fiber reinforced plastic (CFRP) composites and the effects of cryogenic treatment on tensile properties: a comparative study. Compos Part B-Eng.

[33] Song C, Liu Q, Deng S, Li H, Kitamura Y (2019). Cryogenic-based CO_2_ capture technologies: state-of-the-art developments and current challenges. Renew Sust Energ Rev.

[34] Liu J, Guan Z, Li Z (2018). Application of cryogenic and mechanical treatment in degumming of hemp stems. Biosyst Eng.

[35] Guo W, Shao Y, Zhao M, Lü S, Wu S (2020). Varying the treating conditions to rejuvenate metallic glass by deep cryogenic cycling treatment. J Alloys Compd.

[36] Guan Z, Guan Z, Li Z, Liu J, Yu K (2019). Characterization and preparation of nanoporous carbon derived from hemp stems as anode for lithium-ion batteries. Nanoscale Res Lett.

[37] Li Y, Wang F, Liang J, Hu X, Yu K (2016). Preparation of disordered carbon from rice husks for lithium-ion batteries. New J Chem.

[38] Shen X, Cao Z, Chen M, Zhang J, Chen D (2018). A novel flexible full-cell lithium ion battery based on electrospun carbon nanofibers through a simple plastic package. Nanoscale Res Lett.

[39] Geng Q, Tong X, Wenya GE, Yang C, Wang J, Maloletnev AS, Wang ZM, Su X (2018). Humate-assisted synthesis of MoS_2_/C nanocomposites via co-precipitation/calcination route for high performance lithium ion batteries. Nanoscale Res Lett.

[40] Zhang T, Wang F, Zhang P, Wang Y, Chen H, Li J, Wu J, Chen L, Chen ZD, Li S (2019). Low-temperature processed inorganic perovskites for flexible detectors with a broadband photoresponse. Nanoscale Res Lett.

